# Comparison of “Word” vs. “Picture” version of the Free and Cued Selective Reminding Test (FCSRT) in older adults

**DOI:** 10.1016/j.dadm.2014.11.006

**Published:** 2015-03-29

**Authors:** Molly E. Zimmerman, Mindy J. Katz, Cuiling Wang, Leah C. Burns, Robert M. Berman, Carol A. Derby, Gilbert L'Italien, David Budd, Richard B. Lipton

**Affiliations:** a Department of Neurology, Albert Einstein College of Medicine, Bronx, NY, USA; b Department of Psychology, Fordham University, Bronx, NY, USA; c Department of Epidemiology and Population Health, Albert Einstein College of Medicine, Bronx, NY, USA; d Global Health Economics & Outcomes Research, Bristol-Myers Squibb, Lawrenceville, NJ, USA; e Former employee of Global Clinical Research, Bristol-Myers Squibb, Wallingford, CT, USA

**Keywords:** Episodic memory, Older adults, Dementia, Cognitive impairment, Free and Cued Selective Reminding Test

## Abstract

**Background:**

This study examined the psychometric relationship between the Word and Picture versions of the Free and Cued Selective Reminding Test (FCSRT) and developed an equation for score conversion.

**Methods:**

One hundred and eight-seven participants were administered the FCSRT-Picture and FCSRT-Word on two visits using a randomized counterbalanced design.

**Results:**

Participants had a mean age of 82.1 (standard deviation or SD = 5.4) and mean education of 14.5 (SD = 3.3) years. Mean FCSRT-Picture Free Recall score (mean 33.0 years, range: 17–44 years) was 7.9 points higher than the Word score (mean 25.1 years, range: 3–43 years). The Picture and Word FCSRT correlations for Free Recall and Total Recall were r = 0.56, *P* < .01 and r = 0.46, *P* < .01, respectively.

**Discussion:**

The Picture and Word versions of the FCSRT were moderately associated in a sample of cognitively normal older adults. The score mean differences and variability between FCSRT-Picture and FCSRT-Word indicate that their scores should not be considered equivalent.

## 1 Background

Memory is a complex cognitive construct that holds enormous significance for clinicians, researchers, and the general population, particularly older adults. Memory abilities decline over the adult lifespan [1], [2], [3], are a crucial feature of the diagnosis of dementia [4], and critically support the quality of life and activities of daily living [5], [6], [7]. The Free and Cued Selective Reminding Test (FCSRT; [8]) is a neuropsychological test of verbal memory. The FCSRT differs from other tests of episodic memory in that it controls for individual differences in attention and cognitive processing through the implementation of a “controlled learning” study procedure in which the examinee searches for study items (e.g., “horse”) in response to a category cue (e.g., “animal”). The category cues are then used to facilitate the recall of items not retrieved during a free recall test phase of the task. The controlled learning approach of the FCSRT uniquely maximizes encoding specificity and learning through the promotion of deep semantic processing. The FCSRT has been used to identify prevalent dementia, incident dementia, mild cognitive impairment, and distinguish Alzheimer's disease from other types of dementia [9], [10], [11], [12], [13], [14]. We have also shown [15] that the FCSRT has a stronger predictive utility for the identification of individuals who will develop dementia over a 2- to 4-year period compared with a widely used test of episodic memory, the Logical Memory subtest of the Wechsler Memory Scale–Revised [16]. The FCSRT is also a strong correlate of neuroimaging and neuropathological markers [17], [18], [19], [20], [21].

Two versions of the FCSRT differ primarily in terms of their presentation of study material during acquisition. One version uses pictures to depict test items (“Picture version”; e.g., see [11], [22]) while another uses printed words (“Word version”; e.g., see [20], [23]). The picture version was initially reported in 1987 [8] and is based on a selective reminding procedure first described by Buschke and Fuld more than a decade earlier [24]. The word version is a modification of the picture version, perhaps most notably described in a 2007 article by Sarazin and colleagues [12]. The specific motivation for the version modification is unknown, but was likely driven by differences in study setting or researcher preference, as is common in neuropsychological practice with instruments that have a long history. Nonetheless, both picture and word versions are frequently used in current research and clinical settings, yet no study has directly addressed whether they produce equivalent scores. Therefore, the goal of this study was to examine the psychometric relationship between the Word and Picture versions of the FCSRT in a prospective sample of nondemented older adults. In addition, we sought to develop an algorithm for FCSRT score conversions.

## 2 Methods

### 2.1 Study participants

The prospective observational sample consisted of 187 study participants recruited between September 2011 and April 2013 from consecutive visits to the Einstein Aging Study (EAS), an ongoing community-based study of ethnically diverse individuals residing in the Bronx, New York. EAS study design and methods are described in more detail elsewhere [25]. The current substudy was approved by the Albert Einstein College of Medicine Institutional Review Board and informed consent was obtained from all participants. To be eligible for study enrollment, participants were over the age of 70 years, Bronx residents, noninstitutionalized, and spoke English. Exclusion criteria for this study included a previous diagnosis of dementia, visual or auditory impairments that precluded neuropsychological testing, active psychiatric symptomatology that interfered with the ability to complete assessments, or a score of 8/33 or higher on a measure of global cognitive function (Blessed Information-Memory-Concentration test (BIMC [26]). A BIMC score greater than equal 8 is a commonly used cut score for dementia diagnosis, with higher scores on the BIMC reflecting more errors on test items [10]. Participants also completed measures of an estimate of premorbid intellectual ability (Reading subtest from the Wide Range Achievement Test 3 [27]) and depressive symptoms (15-item Geriatric Depression Scale [28]).

### 2.2 Free and Cued Selective Reminding Test—picture version

The FCSRT-Picture procedure consists of two phases; a Study phase and a Test phase. During the Study phase, participants were asked to provide the name of 16 simple line drawings of common recognizable items, each from a different semantic category. In the event that a participant failed to correctly name the item, they were provided with the correct response. Participants were then presented with a 2 × 2 grid containing four of the previously presented simple line drawings. Participants were given a unique category cue (e.g., “animal”) and asked to orally identify the picture of the item that falls within this category (e.g., “horse”). After all four items were correctly identified, the card was removed and immediate cued recall was tested by prompting with the category cue (e.g., “Which one was the animal?”). Participants were reminded of items they failed to retrieve. This procedure was repeated for a total of 16 novel items. During the Test phase, participants were administered three trials of free and cued recall with each proceeded by an interference trial of counting backwards for 20 seconds. The primary variables of interest for the current analyses included the following: (1) Free Recall: For each of three trials, participants freely recalled as many items as possible (score range: 0–48); (2) Cued Recall: Following each Free Recall trial, category cues were provided for items not retrieved during free recall (score range: 0–48); and (3) Total Recall: Free + Cued Recall (score range: 0–48). This test has two equivalent forms and the psychometric properties are described fully in [29], including reliability, essential unidimensionality, and classification accuracy. In this study, picture stimuli were presented via computer screen and the test was administered by a trained research assistant.

### 2.3 Free and Cued Selective Reminding Test–word version (FCSRT-Word)

The FCSRT-Word version consists of an identical administration as the Picture version with two exceptions; (1) the 16 study items were presented as printed words and (2) there was no immediate cued recall procedure (described previously) included in the Study phase. This test has two equivalent forms and was administered by a research assistant using a paper-and-pencil method. The FCSRT-Word was adapted from the original FCSRT-Picture version and to our knowledge, there are no publications that specifically report on the psychometric properties of the Word version. Both the items and semantic categories used in FCSRT-Word differ from those used in FCSRT-Picture.

### 2.4 The Logical Memory I subtest from the Wechsler Memory Scale–Revised [16]

Logical Memory (LM) is a widely used measure of episodic verbal memory that served in this study as well-validated reference point for the FSCRT scores. In the LM task, participants hear two contextually related short stories and are immediately asked to recall details of each story. Scores range from 0 to 50.

### 2.5 Study procedures

One hundred and eight-seven participants were administered the FCSRT-Picture and FCSRT-Word on two separate clinic visits using a randomized counterbalanced design. The free recall scores were used to develop the algorithm to predict FCSRT-Word from FCSRT-Picture scores as this score has been shown to be the most predictive of the development of dementia and cognitive impairment [10], [11], [30]. A subset of participants was administered both versions of the FCSRT-Picture (n = 20) or FCSRT-Word (n = 20) on both clinic visits in a randomized counterbalanced design to establish test-retest reliability of the two versions of the FCSRT. Participants in this subsample received the equivalent forms of each version of the FCSRT to control for internal validity threats such as learning and history.

### 2.6 Statistical analyses

The reliability of the FCSRT picture and word scores were assessed by intraclass correlation coefficients (ICC) estimated from linear mixed effects models using the test-retest subsamples. Linear regression models were used to determine the association of scores on FCSRT-Picture and FCSRT-Word, especially the prediction of FCSRT-Word given FCSRT-Picture, without consideration of the measurement error in FCSRT-Picture and FCSRT-Word. The association between the true scores of the two versions of FCSRT was also calculated using the test-retest reliability information to take measurement error into account. Pearson correlation coefficients were used to evaluate the relationship between performance on the two versions of the FCSRT and the LM I subtest. All tests were two sided and *P* values of less than .05 were considered statistically significant.

## 3 Results

### 3.1 Sample characteristics

Demographics and sample characteristics are shown in Table 1.

### 3.2 Test-retest reliability

Test-retest reliability based on ICC calculated from linear mixed effects models was good for both the FCSRT-Picture Free Recall (0.75) and FCSRT-Word Free Recall (0.80). Test-retest reliability was 0.69 for FCSRT-Picture Total Recall and 0.83 for FCSRT-Word Total Recall. However, because of ceiling effects for the Total Recall score for both FCSRT-Picture and FCSRT-Word, these results should be considered with caution.

### 3.3 FCSRT-Picture and FCSRT-Word relationship

**Fig. 1:**
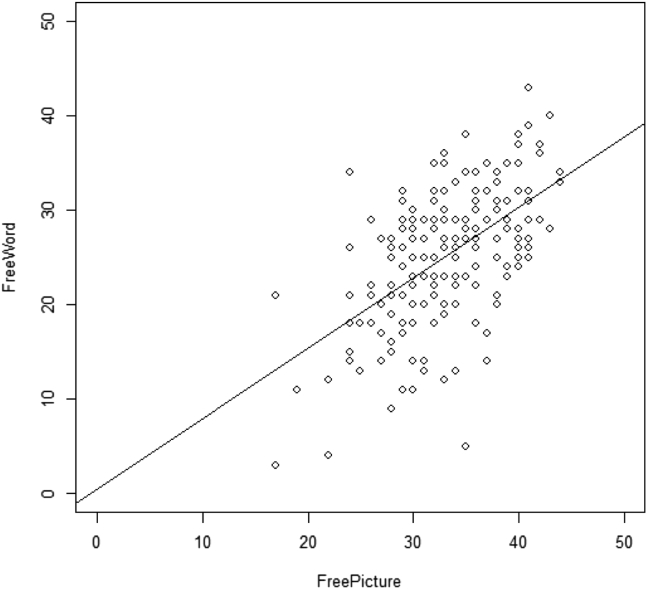
Relationship between free recall scores on Free and Cued Selective Reminding Test (FCSRT)-Picture and FCSRT-Word with regression line.

The mean FCSRT-Picture Free Recall score (mean 33.0, range: 17–44 years) was 7.9 points higher than the Word score (mean 25.1, range: 3–43 years) (see Table 1) indicating that the test procedures on FCSRT-Picture produced higher levels of free recall. The observed Pearson correlation between the Picture and Word FCSRT Free Recall was 0.56 (see Fig. 1; *P* < .01). After correcting for test-retest reliability, the unattenuated correlation between the true Picture and Word FCSRT Free Recall scores was 0.74 (*P* < .01).

The mean FCSRT-Picture Total Recall score (mean 47.7, range: 39–48 years) was 4.3 points higher than the Word score (mean 43.4, range: 16–48 years). The observed Pearson correlation between the Picture and Word FCSRT Total Recall was 0.46 (*P* < .01). After correcting for test-retest reliability, the unattenuated correlation between the true Picture and Word FCSRT Total Recall scores was 0.62 (*P* < .01). Ceiling effects for the Total Recall score for both FCSRT-Picture and FCSRT-Word indicate that these results should be considered with caution.

### 3.4 Regression of FCSRT-Word on FCSRT-Picture

FCSRT Free Recall scores were used to develop the algorithm to predict FCSRT-Word from FCSRT-Picture scores as this score has been shown to be the most predictive of the development of dementia and cognitive impairment [10], [11], [30]. Although a linear trend appeared present in the scatter plot for the relationship between FCSRT-Picture and Word Free Recall scores (Fig. 1), we tested the possible quadratic trend and it was ignorable (estimate = −0.0071, *P* = .53). Based on the linear regression model using the observed FCSRT-Picture and Word Free Recall scores, the formula for predicting a mean FCSRT-Word Free Recall score using a FCSRT-Picture Free Recall score after considering age, education, gender, and ethnicity was:

W=14.12+0.72∗P+1.33∗Gender+0.11∗Education−0.18∗Age−1.17∗Ethnicity

where *W* = FCSRT-Word Free Recall score, *P* = FCSRT-Picture Free Recall score, *Gender* is 0 = male and 1 = female, *Education* is years of education, *Age* is in years, *Ethnicity* is 0 = Caucasian and 1 = non-Caucasian.

The R^2^ of the model is 0.35. The predicted residual sum of square as leave-one-out cross-validation of the model is 6462.93 (average 34.56). As *Age* was the only significant covariate in this model, a reduced model was calculated in which mean FCSRT-Word Free Recall score was predicted using FCSRT-Picture Free Recall score and *Age*:

W=15.16+0.73∗P−0.17∗Age

The R^2^ of this reduced model was 0.33. An F-test for model comparison between the full and reduced models resulted in *F*_3,181_ = 1.42, *P* = .24, indicating that the models did not differ.

### 3.5 Relationships between FCSRT versions and Logical Memory I subtest

Pearson correlation coefficients revealed that higher scores on LM were significantly associated with higher scores on FCSRT-Picture Free Recall (r = 0.27, *P* < .01) and FCSRT-Word Free Recall (r = 0.36, *P* < .01).

## 4 Discussion

We found that the Picture and Word versions of the FCSRT were moderately associated in a sample of cognitively normal older adults. The score mean differences and variability between FCSRT-Picture and FCSRT-Word indicate that scores on each version should not be considered equivalent. We provide two formulas to facilitate the conversion of free recall scores between the two versions among nondemented older adults. One formula includes the consideration of age, gender, education, and ethnicity whereas a simplified model considers only age. Statistical comparison of these alternative models indicates that differences in model fit are negligible; however, verification in a larger independent sample may be warranted.

Although both versions are commonly used, the FCSRT-Picture [11], [22] and FCSRT-Word [20], [23] differ from one another in several key ways and no study has directly addressed the equivalency of scores between the two versions. The awareness of version differences among those in the research and clinical arenas may be low due to the fact that many published studies reporting FCSRT data do not specify the employed version [21], [31]. In addition, even within versions, variation in test administration exists in the literature. For example, recent reports of the FCSRT-Picture note three trials of free and cued recall whereas earlier reports note four trials [30]. A delayed free and cued recall trial following a 30-minute delay has also been reported [32], although most studies do not apply a delayed recall condition. Another variation is the inclusion or omission of the immediate cued recall procedure in which the participant is presented with the semantic cues and test items during acquisition and then immediately presented with only the cues while being asked to provide the associated test items (e.g., “Which one was the animal?”). Notwithstanding these relatively minor modifications, a critical administration difference lies in the presentation of the test items during acquisition; that is, either as pictures or words. The FCSRT-Picture was initially developed by Grober and colleagues [8] and was itself an adaptation of the selective reminding procedure originally described by Buschke and Fuld in 1974 [24]. In the FCSRT-Picture, test items are depicted pictorially as simple black and white line drawings. The FCSRT-Word was modified from the Picture version and depicts the test items as printed words that are read aloud by the participant. Although data from both versions have been shown to robustly identify prevalent dementia, incident dementia, mild cognitive impairment, and distinguish Alzheimer's disease from other types of dementia [9], [10], [11], [12], [13], [14], [30], comparisons of findings across studies and agreement on appropriate clinically significant cut-scores have been challenging given the apparent differences in scores obtained from each version. For instance, a cut-score of 24/48 free recall for the prediction of the development of dementia has been reported for FCSRT-Picture [8] although a cut-score of 17/48 free recall has been reported for FCSRT-Word [12]. This study directly addressed this issue by examining the relationship between the two versions of this test. Although score conversions vary depending on demographic variables, our results suggest that the previously reported cut-score of 24/48 free recall FCSRT-Picture would be converted to a score of 18/48 free recall FCSRT-Word for an 85-year-old and 21/48 free recall FCSRT-Word for a 70-year-old community-dwelling adult.

On average, we found that scores were higher on FCSRT-Picture compared with the FCSRT-Word version. The relative enhancement of performance with pictorially presented information is consistent with the Pictorial Superiority Effect, a well-described finding among healthy younger and older adults [33], [34], [35] and older adults with mild cognitive impairment [36]. Three main theories have been proposed for why pictures are generally better remembered than words. The dual-coding [37] theory states that during the acquisition of information, pictures elicit both a verbal and visual representation although words only elicit a verbal representation in the memory process. Thus, pictures produce better performance because there are twice as many representations to facilitate recall and recognition. The distinctiveness hypothesis posits that pictures contain a wealth of distinctive visual features that are absent in the presentation of words, thus facilitating encoding and retrieval [38]. Finally, the semantic processing hypothesis suggests that pictures allow elaborate conceptual and perceptual processing of test items that words are not able to provide [39]. Although our study was not designed to examine the mechanisms of the Pictorial Superiority Effect, the wealth of literature that has replicated this finding lends strong support to the mean differences between the FCSRT-Picture and FCSRT-Word scores that we observed. As the graph (see Fig. 1) shows, however, some older adults performed better on the Word version than the Picture version; this is consistent with individual differences that are commonly observed in behavioral testing and suggests that the Pictorial Superiority Effect is not uniformly operative in all individuals. In addition, it is important to note that higher scores do not necessarily advocate use of one version over the other. However, caution should be taken when comparing results across studies with special consideration of version type.

There are several limitations to this study that warrant further discussion. Our study design focused on two specific versions of the FCSRT that differed as a function of the presentation of the study items. As noted previously, however, there are additional modifications in task administration whose examination was beyond the scope of this investigation. Our decision to focus broadly on differences between picture vs. word study items was driven by the preponderance of recent research reports using the FCSRT that have differed largely by the mode of presentation of the to-be-learned information. Another consideration for the interpretation of our findings is that a commonly used measure of episodic memory, the Logical Memory I subtest, was only modestly associated with performance on the FCSRT-Picture and FCSRT-Word (r = 0.27 and r = 0.36, respectively). These relationships highlight the multifaceted nature of the episodic memory construct and suggest that the administration of multiple tests that interrogate various aspects of episodic memory may be necessary to fully capture the complexity of memory abilities in humans. A final limitation is that our sample did not include individuals who are at a high risk for or with Alzheimer's disease or other dementias. As recent research trends have focused on early detection of prodromal dementia, we elected to restrict our sample to nondemented older adults who live independently in the community. Therefore, our findings are not generalizable to older adults with dementia or individuals specifically seeking treatment for memory concerns, such as those evaluated at a memory disorder clinic. Future studies should seek to directly compare the sensitivity of each version of the FCSRT to the early identification of memory decline or dementia.

In summary, our data strongly suggest that data from two commonly used versions of the FCSRT, the FCSRT-Picture and FCSRT-Word, should not be considered equivalent. Differences between the two versions are likely because of the Pictorial Superiority Effect which confers a performance advantage when test stimuli are presented as pictures. Future studies may expand the application of these findings to older adults with dementia. In addition, future studies may wish to investigate implications of the Pictorial Superiority Effect within the context of FCSRT-Picture administration to older adults at a high risk for dementia.

**Research in context**

1. Systemic review: A literature review using the search engine PubMed was conducted using the search term “Free and Cued Selective Reminding” for all years of publication. Snowballing techniques were also used to identify relevant citations.
2. Interpretation: We found that the Picture and Word versions of the Free and Cued Selective Reminding Test (FCSRT) were moderately associated in a sample of cognitively normal older adults. The score mean differences and variability between FCSRT-Picture and FCSRT-Word indicate that their scores should not be considered equivalent. The relative enhancement of performance with pictorially presented information is consistent with other reports that pictures are generally more easily remembered than words.
3. Future directions: Future studies should directly compare the predictive utility for each FCSRT version for the diagnosis of mild cognitive impairment and dementia. In addition, the psychometric properties of each FCSRT version should be evaluated in older adults with mild cognitive impairment and dementia.

## Figures and Tables

**Table 1:** Demographics and sample characteristics (N = 187)

	Mean	SD
Age (yrs)	82.1	5.4
Education (yrs)	14.5	3.3
Gender (women)∗	121	64.7
Ethnicity (Caucasian)∗	115	61.5
BIMC (total scores 0–33)	1.4	1.7
WRAT 3 reading subtest (Grade equivalency)	11.8	2.3
GDS-15 item (total score 0–15)	1.7	2.1
English First Language (yes)∗	146	78.1
FCSRT-Picture Free Recall Score (0–48)	33.0	5.4
FCSRT-Word Free Recall Score (0–48)	25.1	7.1
FCSRT-Picture Total Recall Score (0–48)	47.7	1.0
FCSRT-Word Total Recall Score (0–48)	43.4	5.4
LM I subtest (total score 0–50)	22.3	7.4

Abbreviations: SD, standard deviation; BIMC, Blessed-Information-Memory-Concentration test; WRAT 3, Wide Range Achievement Test version 3; GDS, Geriatric Depression Scale; FCSRT, Free and Cued Selective Reminding Test; LM I, Logical Memory I from the Wechsler Memory Scale—Revised.

∗ Presented as N, %.
